# Preparation and Characterization of Water-Insoluble Gardenia Blue Pigment

**DOI:** 10.3390/ma14216594

**Published:** 2021-11-02

**Authors:** Yakun Gao, Jinchuan Xu, Guorong Liu, Rong Nie, Jiaojiao Duan, Duoxia Xu, Chengtao Wang

**Affiliations:** 1 Beijing Advanced Innovation Center for Food Nutrition and Human Health, Beijing Engineering and Technology Research Center of Food Additives, Beijing Technology and Business University, Beijing 100048, China; gaoyakun2017@126.com (Y.G.); nierong@st.btbu.edu.cn (R.N.); duan.jiaojiao@st.btbu.edu.cn (J.D.); xuduoxia@th.btbu.edu.cn (D.X.); wangchengtao@th.btbu.edu.cn (C.W.); 2 Tianjin Heping District Market Supervision and Management Bureau, Tianjin 300041, China; 3 School of Food Science and Engineering, South China University of Technology, Guangzhou 510640, China; jinchuanxu@foxmail.com

**Keywords:** gardenia blue pigment, water-insoluble, structure study, solubility study, stability study

## Abstract

Based on molecular simulations, the synthetic route of water-insoluble gardenia blue pigment was prepared by the reaction of genipin and L-Phenylalanine methyl ester hydrochloride. A highly purified pigment was obtained after extraction by chloroform and purification by silica gel column chromatography, and the value of color is up to 288. A study on the structural characteristics of the pigment was implemented with a scanning electron microscope, ultraviolet-visible spectrophotometer, Fourier transform infrared spectrometer, X-ray photoelectron spectrometer, and quatropde-time of flight mass spectrometer. The results showed that the surface of the pigment was largely smooth and spherical; The *λ*_max_ was 607 nm, and the main functional groups include O-C=O, C=O, C-N, C=C, OH, and benzene ring; We detrained six different molecular weight and chemical structures of pigments and speculated the particular structures and formation mechanisms of three kinds of pigment, whose molecular weights are 690.1156, 720.1226, and 708.1246 Da, respectively. The pigment was only able to be dissolved in ethanol, methanol, acetone, ethyl acetate, and other strong polar organic solvents, but was not able to be dissolved in water, ethyl ether, petroleum ether, and other weak polar organic solvents. In terms of light and thermal stabilities, water-insoluble gardenia blue pigment is significantly better than water-soluble gardenia blue pigment (*p* < 0.05). When it is under direct light for 7 days or incubated at 80–120 °C for 24 h, the pigment residual rates were 74.90, 95.26, 88.27, and 87.72%, respectively.

## 1. Introduction

Pigments are widely used in food, medicine, maquillage, and other fields. Due to consumers’ concern about the safety of synthetic pigments, the application of synthetic pigments was confined to a limited amount and natural pigments are used more frequently. The blue pigments used so far have mainly been synthetic and the food industry has expressed a growing interest in the use of natural blue pigment, especially for confectionery and pastries [1,2].

Gardenia blue pigment is a natural blue pigment of food additives [3]. The gardenia blue pigment is prepared by primary amine compounds and genipin from the hydrosylate of jasmine, which is colorful, safe, and has strong coloring power [4,5,6,7]. The gardenia blue pigment is always water-soluble, but it is unable to be dissolved in organic solvents and oil. These features limit the scope of application of the gardenia blue pigment. We also need some water-insoluble blue pigments for some necessary uses. Some researchers have already obtained some hydrophobic gardenia blue pigments through esterification or phospholipid acylation [8,9]. Some researchers studied the reaction mechanism of the hydrophobic gardenia blue pigment by its intermediates [10,11,12,13]. However, due to their high polymerization, the water-soluble and hydrophobic gardenia blue pigments have no exact structure and molecular weight [14,15,16].

The stability of the natural pigments is challenged due to light, heat, pH, and the existence of metal ions or oxidants and reductants. Some researchers have studied the stability of water-soluble gardenia blue pigment, and they found that it was most unstable under light [17,18,19]. Others tried to improve the pigments’ stability by adding color fixative, embedding and chemical modifying, and other different kinds of methods [20,21,22,23].

In order to widen the melting range and increase the stability of the gardenia blue pigment, we prepared a new kind of water-insoluble gardenia blue pigment and studied its structure and molecular weight preliminarily.

## 2. Experiment

### 2.1. Materials

Genipin (Linchuan zhixin Biotechnology Co., Ltd., Jiangxi, China). L-Phenylalanine methyl ester hydrochloride (Shanghai yuanye Biotechnology Co., Ltd., Shanghai, China). HPLC Acetonitrile (CH_3_CN) (Thermo Fisher Scientific (China) Co. Ltd., Shanghai, China). Stearyl chloride (Shanghai Macklin Biochemical Co., Ltd., Shanghai, China). Silica gel plate GF254, Glass chromatography jar, 200–300 mesh column chromatography silica gel (Qingdao Marine Chemical Co., Ltd., Shandong, China). Mineral ether (Beijing Chemical Works, Beijing, China). pH was adjusted by pH400 (Alalis, Shanghai, China). Color properties were performed on a CM-3600A Color tester (Konica Minolta, Tokyo, Japan). The microstructure was observed on an S-4800 field emission scanning electron microscope (FE-SEM) (Hitachi, Tokyo, Japan). Ultraviolet-visible (UV-vis) spectra were recorded on a UV-2450 spectrophotometer (Shimadzu Corporation, Kyoto, Japan). Functional groups were determined by AVATAR 370 Fourier transform infrared spectrometer (FTIR) (Thermo Nicolet Corporation, Madison, WI, USA) and Thermo esca lab 250Xi X-ray photoelectron spectrometer (XPS) (Thermo Fisher, Waltham, MA, USA). Liquid chromatography-mass spectrometry (LC-MS) analysis was performed on a 6530 LC quatropde-time of flight mass spectrometer (Q-TOF LC/MS) (Agilent, California, CA, USA) using the following column: Inertsil ODS-4: 4.5 mm × 150 mm, 5 μm (Agilent, Philadelphia, PA, USA).

### 2.2. Synthesis of Water-Insoluble Gardenia Blue Pigment

To a solution of genipin (2.5 mmol) and phenylalanine methyl ester hydrochloride (3.0 mmol) in ethanol (50 mL) NaOH was added to pH = 7.0. After stirring for 25 h in aerobic condition at 60 °C, the reaction mixture was distilled by a rotary evaporator and dried [11]. Then the reaction mixture was dispersed in ultrapure water and extracted with chloroform by 3 times and dried by sodium sulfate (Na_2_SO_4_). Then the water-insoluble gardenia blue pigment was filtrated and concentrated by using silica gel column chromatography (ethyl acetate).

### 2.3. UV-Vis Detection Method

The water-insoluble gardenia blue pigment was dissolved by ethanol. The wavelength was 400–700 nm.

### 2.4. FTIR Detection Method

Prepare the samples: The mass ratio of water-insoluble gardenia blue pigment and potassium chloride was 1:100, mixed them well and grind to 100 mesh. Then take a small amount of mixture to flake. Instrument parameters are as follows: DTGS detector, the spectrum range was 4000–400 cm^−1^ and the resolution ratio was 4 cm^−1^. Take off the water and air disturbance when scanning.

### 2.5. XPS Detection Method

XPS was measured to determine the C, N, and O atoms of water-insoluble gardenia blue pigment at room temperature [24]. Instrument parameters: excitation source (Monochromatic Al K_α_, HV = 1486.6 eV), the capacity was 150 W, the beam spot was set at 650 *μ*m, the measurement accuracy was set at 0.1 eV and the vacuum reached 10^−9^ Pa. We used C1s = 284.8 eV to correct charge.

### 2.6. Thin Layer Chromatography (TLC)

The developing agent was a chloroform/ethanol mixture (22:1, v/v). The R_f_ value of each pigment spot was calculated [25,26].

### 2.7. LC-MS Detection Method

LC-MS was used to test the molecular mass of gardenia blue pigments. The instrument conditions were as follows, the sample temperature was kept at 25 °C, a 5.0 *μ*L sample was injected, the flow rate was 0.2 mL/min, 2% methanoic acid-acetonitrile (A) and 2% methanoic acid-water (B) were the elution, the gradient of elution was: 0–40 min (4%–100% A); 40–50 min (100% A); 50–52 min (4–100% A); 52–60 min (4% A). In addition, nitrogen gas was regarded as the nebulizer and auxiliary, which the desolvation temperature, pressure, and the flow rate was set at 300 °C, 35 psig, and 600 L/h. The argon gas was considered as the collision, and the energy was STE at 50 eV. The ESI capillary voltage was 4 kV. The mass scanned area was *m*/*z* 100–1500.

### 2.8. Solubility of the Pigment

Soluble equal weight: The water-soluble and water-insoluble gardenia blue pigment were put into the different same volume solvents.

### 2.9. The Light and Thermal Stabilities of the Pigments

The light stability of the pigment was evaluated at different light conditions, sunlight (40,000 lux), lamplight (13,000 lux), room light (300 lux), and dark (control group). The thermal stability of the pigment was tested at different temperatures, 4 °C, 40 °C, 60 °C, 80 °C, 100 °C, 120 °C, and room temperature (25 °C, control group). The samples were prepared by dissolving the water-soluble and water-insoluble gardenia blue pigment into ultrapure water and ethanol respectively, then adjusting the absorbance to 0.8. The samples were put at each light condition for 7 days and each temperature for 24 h in glass test tubes and their color properties were identified [1].

### 2.10. Calculation of Chromatism

The color value of the pigment was evaluated by the following equation [27]: $E_{1cm}^{1\%}=\frac{AV}{100m}$. In this equation, $E_{1cm}^{1\%}$ refers to the cuvette (1 cm × 1 cm) and the concentration of the pigment solution is 1%; *A* means the absorbance at the maximum absorption wavelength (*λ*_max_) of the pigment solution; *V* (mL) means the volume of the pigment solution; *m* (g) meansthe quality of the pigment. The color properties of the pigment were evaluated in terms of the CIE L*a*b* system [28,29,30]. Where L* is the brightness, a* is the green (-) or red (+), and b* represents the blue (-) or yellow (+). Chroma parameter (*C*) is defined as $C_{ab}^{*}=\sqrt{a^{*2}+b^{*2}}$, which is the color saturation of the pigments. The color difference ($\Delta L^{*},\;\Delta a^{*},\;\Delta b^{*},\;\Delta C_{ab}^{*},\;\mathrm{and}\;\Delta E_{Lab}^{*}$) was analyzed by using the following equations:

(1) $$\Delta L^{*}=L_{after}^{*}-L_{before}^{*}$$

(2) $$\Delta a^{*}=a_{after}^{*}-a_{before}^{*}$$

(3) $$\Delta b^{*}=b_{after}^{*}-b_{before}^{*}$$

(4) $$\Delta C_{ab}^{*}=C_{after}^{*}-C_{before}^{*}$$

(5) $$\Delta E_{Lab}^{*}=\sqrt{\Delta L^{*2}+\Delta a^{*2}+\Delta b^{*2}}$$

## 3. Results and Discussion

### 3.1. Establishment of Molecular Model of Water-Insoluble Gardenia Blue Pigments

**Figure 1:**
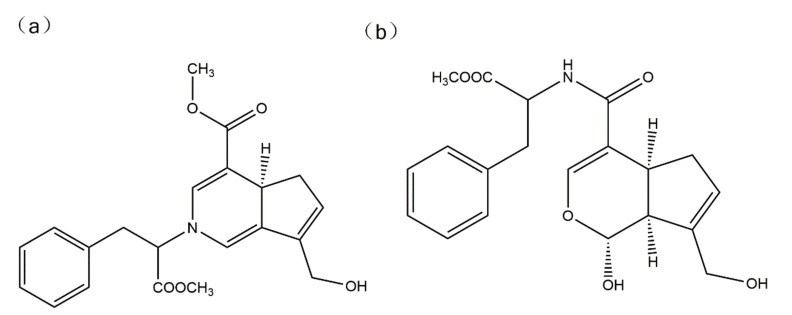
Simulation of the chemical structural formula of the intermediate product 1 (**a**) and intermediate product 2 (**b**) of the water-insoluble gardenia blue pigment.

The simulation of the chemical structural formula of the intermediate products of the water-insoluble gardenia blue pigments was established by ChemBio3D Ultra11.0 (Figure 1). Then the lowest molecular energy conformation model was obtained by HyperChem Release 7.0, the lowest molecular energies of the intermediate product 1 and intermediate product 2 were 23.41 kcal/mol and 21.87 kcal/mol respectively. They are the most stable conformation.

### 3.2. Purification of Water-Insoluble Gardenia Blue Pigment

After extraction by chloroform and purification by silica gel column chromatography, the color value of water-insoluble gardenia blue pigment was up to 288, which was 1.32 times higher than at the beginning (Table 1). It indicates that the purity of water-insoluble gardenia blue pigment becomes higher. The pigment powder is in dark blue, and the pigment solution is in bright blue (ethanol as solvent). The a* (−) of water-insoluble gardenia blue pigment increased, it means the green chromaticity of the pigment reduced, the b* (−) of water-insoluble gardenia blue pigment reduced, it means the blue chromaticity of the pigment increased. These results indicate that water-insoluble gardenia blue pigment becomes bluer.

### 3.3. Microstructure of Water-Insoluble Gardenia Blue Pigment

**Figure 2:**
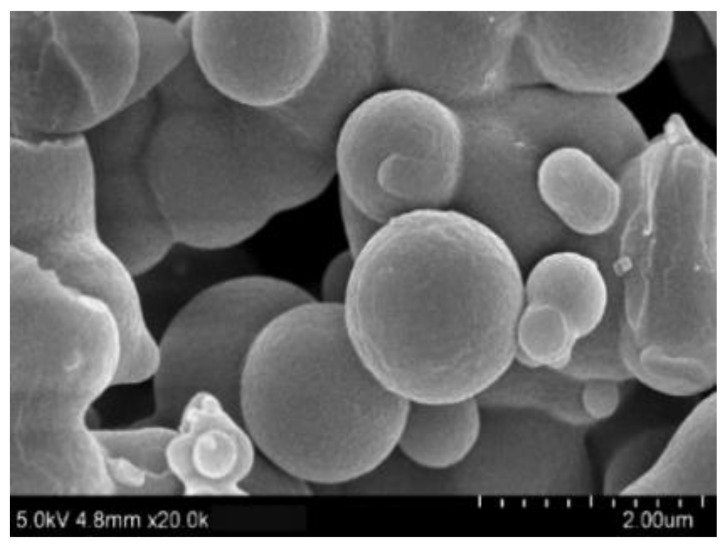
The SEM of water-insoluble gardenia blue pigment.

We studied the microstructure of water-insoluble gardenia blue pigment by FE-SEM by magnifying by 20,000. The results showed that the surface of the water-insoluble gardenia blue pigment was largely smooth and spherical (Figure 2). It makes the water-insoluble gardenia blue pigment hydrophobic.

### 3.4. UV Absorbance Wavelength of Water-Insoluble Gardenia Blue Pigment

**Figure 3:**
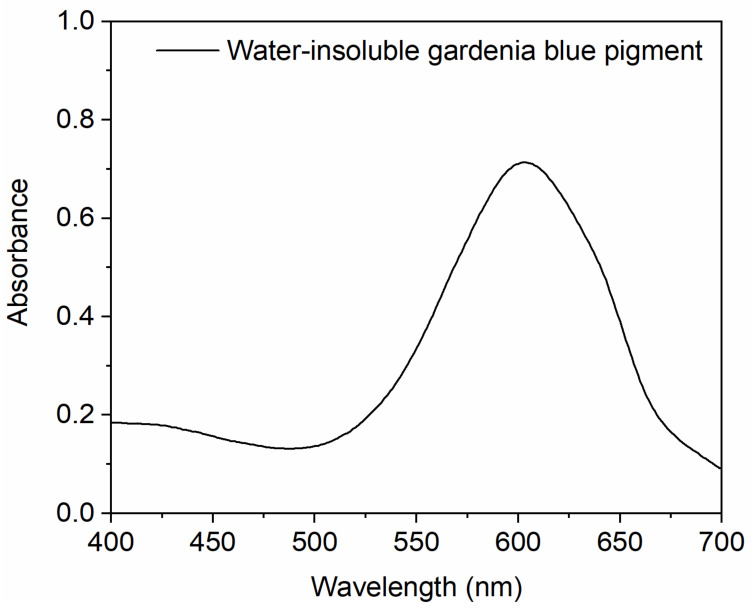
The UV-vis of water-insoluble gardenia blue pigment.

Next, we studied the characteristics of absorbance wavelength of gardenia blue pigments by UV-vis. The blue broad optical absorption band of the pigments is at wavelengths between 590 and 620 nm. Figure 3 shows that the water-insoluble gardenia blue pigment has one sharp absorption peak at 607 nm (*λ*_max_). Masaaki TAKAMI and Yukio SUZUKI ADE made a kind of hydrophobic blue pigment by phosphatidyl genipin and L-Phenylalanine methyl ester hydrochloride, it showed *λ*_max_ at 615 nm in chloroform [8]. It indicates that we got a new water-insoluble gardenia blue pigment.

### 3.5. The Characteristic Function Group Analysis of Water-Insoluble Gardenia Blue Pigment

**Figure 4:**
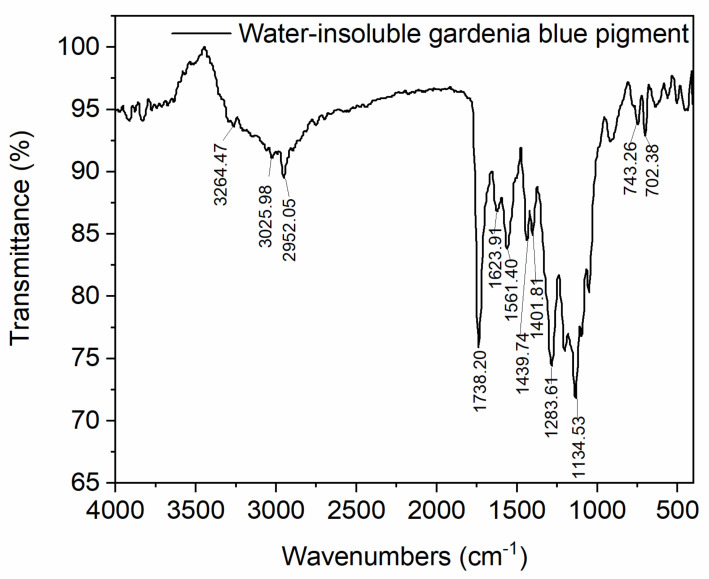
The FTIR of water-insoluble gardenia blue pigment.

Study on the characteristic functional groups of water-insoluble gardenia blue pigments by FTIR and XPS. The FTIR characteristic peaks were showed in Figure 4: 3264.47 (OH); 2952.05 (CH); 1738.20 (C=O); 1623.91, 1561.40 (C=C); 1283.61, 1134.53 (COOC); 3025.98, 1439.74, 1401.81 (aromatic); 702.38, 743.26 (mono-substituted benzene ring) cm^−1^. The results showed that the characteristic absorption peaks of CH, OCH_3_, C=O, COOC, C=C, and aromatic can correspond to the FTIR spectra of genipin and L-Phenylalanine methyl ester hydrochloride [2]. The characteristic absorption peaks of OH became weak due to the lower proportion of the OH group in the pigment.

**Figure 5:**
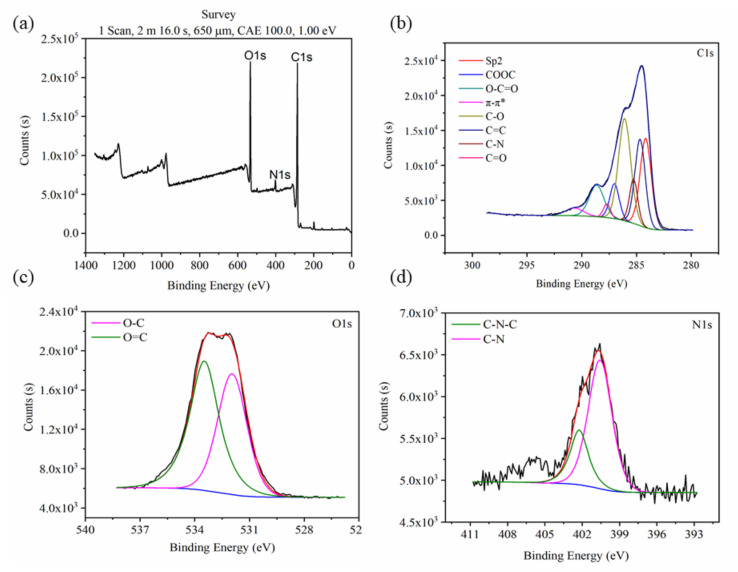
The full-spectrum (**a**), C1s-spectrum (**b**), O1s-spectrum (**c**), and N1s-spectrum (**d**) XPS of water-insoluble gardenia blue pigment.

The results of the full-spectrum XPS of water-insoluble gardenia blue pigment showed that the major elements of water-insoluble gardenia blue pigment are C, N, and O, and the content of C and O are higher than that of N (Figure 5a). The method of separating overlapped curves was adopted to analyze C1s, O1s, and N1s peaks (Figure 5b–d). The contents of the functional groups are in Table 2, Table 3 and Table 4. The C-1s spectrum of water-insoluble gardenia blue pigment presents main peaks at 284.16 eV, 284.68 eV, 286.09 eV related to Sp2 hybrid, C=C, and C-O structures. The O-1s spectrum of water-insoluble gardenia blue pigment presents two peaks at 531.95 eV and 533.47 eV related to O-C and O=C structures. The N-1s spectrum of water-insoluble gardenia blue pigment presents two peaks at 402.24 eV and 400.57 eV related to C-N-C and C-N structures. As a result, XPS shows that water-insoluble gardenia blue pigment has a π-π * resonance system, and functional groups are O-C=O, C=O, COOC, C-N, C=C, and benzene ring.

### 3.6. TLC Separation of Water-Insoluble Gardenia Blue Pigments

Six different pigments were obtained after the separation of the water-insoluble gardenia blue pigments by thin-layer chromatography (chloroform ethanol 22:1). The R_f_ values of varying water-insoluble gardenia blue pigment are recorded in Table 5.

### 3.7. Mass Spectrometry Study of Water-Insoluble Gardenia Blue Pigments

The positive-ion mode of ESI mass spectra was performed to investigate the structures of water-insoluble gardenia blue pigments, which were coded as follows, Z-1, Z-2, Z-3, Z-4, Z-5, and Z-6.

The first-order mass spectra of the six different water-insoluble gardenia blue pigments Z-1–Z-6 showed the molecular weights of the pigments. The [M + Na]^+^ ion was selected as the precursor ion in the MS/MS experiment to give fragmentation information. Table 6 shows the mass and composition of the Na^+^ adduct ions [M + Na]^+^ and main compound ions of the pigments.

**Figure 6:**
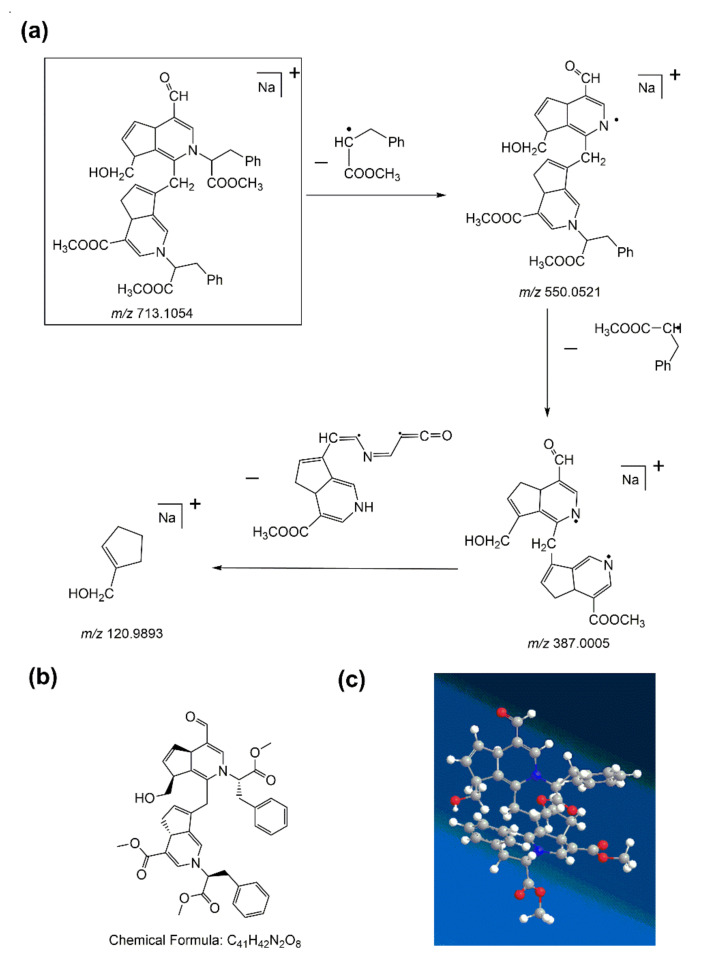
The fragmentation pathway of water-insoluble gardenia blue pigment Z-1 (**a**). The planar and 3D molecular structure simulation graphs of the pigment Z-1 (**b**) and (**c**) (C-gray, O-red, N-blue, H-white).

In the first-order MS of water-insoluble gardenia blue pigment Z-1, the [M + Na]^+^ ion at *m/z* 713.1054 was studied as the base peak, which proved that the formula weight of the water-insoluble gardenia blue pigment Z-1 is 690.1156 Da (the formula weight of sodium (Na) is 22.9898 Da). The precursor ion ([M + Na]^+^) in the MS/MS test could offer varying fragmentation information. The product ion (*m/z* 550.0521) had the highest abundance and the product (*m/z* 387.0005) corresponded to the loss of two Δm = 163 fragments of L-Phenylalanine methyl ester hydrochloride. More peaks simultaneously appeared at *m/z* 120.9893 because of ring cleavage, which corresponded to the loss of a Δm = 266 fragment. The fragmentation pattern of water-insoluble gardenia blue pigment Z-1 is shown in Figure 6a. The structure of water-insoluble gardenia blue pigment Z-1 had significantly better functional substituents and the cleavage of the basic skeleton of the product. Then we can also deduce that the chemical formula of water-insoluble gardenia blue pigment Z-1 is C_41_H_42_N_2_O_8_. The planar and 3D molecular structure simulation graphs are shown in Figure 6b,c.

**Figure 7:**
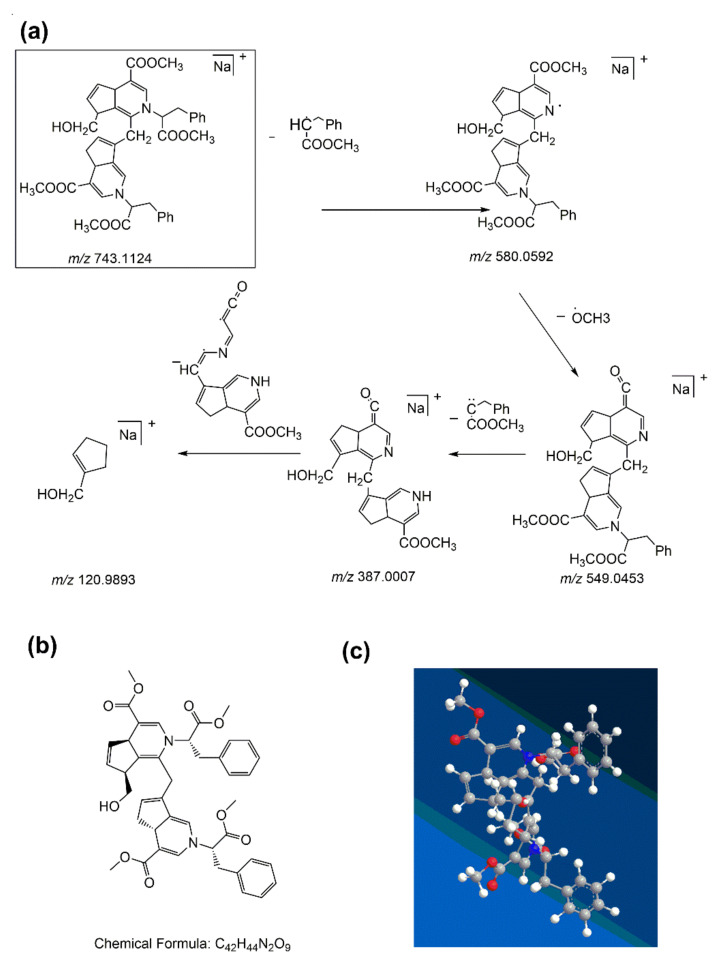
The fragmentation pathway of water-insoluble gardenia blue pigment Z-2 (**a**). The planar and 3D molecular structure simulation graphs of the pigment Z-2 (**b**) and (**c**) (C-ray, O-red, N-blue, H-white).

In the first-order MS of water-insoluble gardenia blue pigment Z-2, the [M + Na]^+^ ion at *m/z* 743.1124 was researched as the base peak, which proves the formula weight of the water-insoluble gardenia blue pigment Z-2 is 720.1226 Da. The precursor ion ([M + Na]^+^) in the MS/MS test could offer more fragmentation information. The product ion is at *m/z* 580.0592 with low abundance, and it corresponded to the loss of a Δm = 163 fragment of L-Phenylalanine methyl ester hydrochloride. It is similar to water-insoluble gardenia blue pigment Z-1. With the loss of -OCH_3_ (Δm = 31), further peaks simultaneously appeared at *m/z* 549.0453, which was the highest abundance. The fragment ion at *m/z* 387.0007 was produced by the loss of a Δm = 162 fragment of L-Phenylalanine methyl ester hydrochloride. As water-insoluble gardenia blue pigment Z-1, further peaks simultaneously appeared at *m/z* 120.9893 because of ring cleavage, and it corresponded to the loss of a Δm = 266 fragment. The fragmentation pattern of water-insoluble gardenia blue pigment Z-2 is shown in Figure 7a. The structure of water-insoluble gardenia blue pigment Z-2 can be deduced by the functional substituents and the cleavage of the basic skeleton of the product. In this way, we can deduce that the chemical formula of water-insoluble gardenia blue pigment Z-2 is C_42_H_44_N_2_O_9_. The planar and 3D molecular structure simulation graphs are shown in Figure 7b,c.

**Figure 8:**
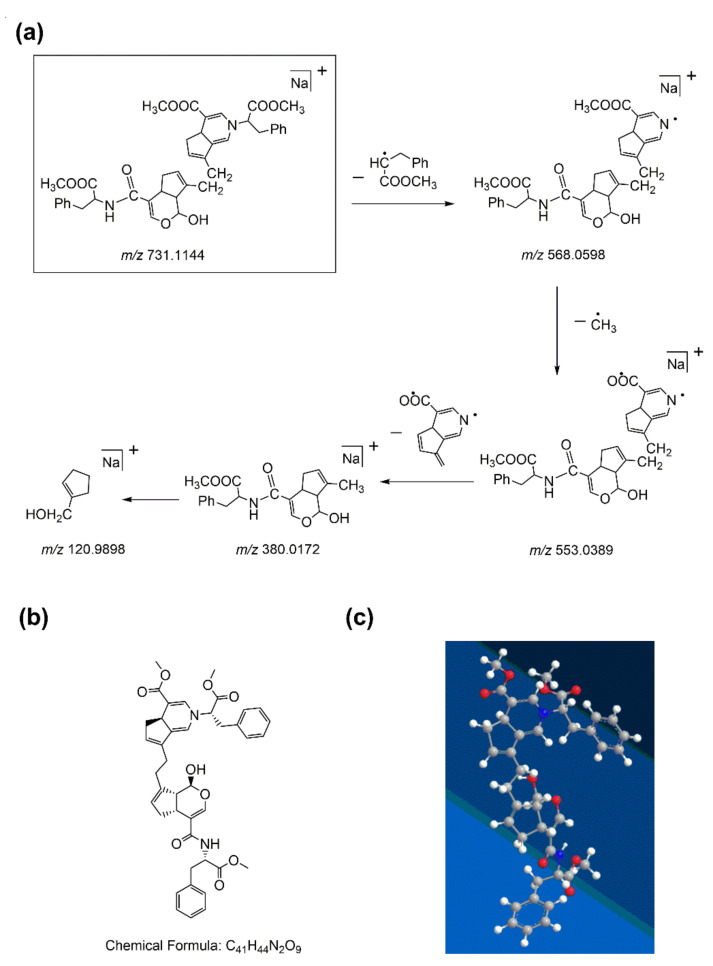
The fragmentation pathway of water-insoluble gardenia blue pigment Z-3 (**a**). The planar and 3D molecular structure simulation graphs of the pigment Z-3 (**b**) and (**c**) (C-gray, O-red, N-blue, H-white).

In the first-order MS of water-insoluble gardenia blue pigment Z-3, the [M + Na]^+^ ion at *m/z* 731.1144 was tested as the base peak which proves the formula weight of the water-insoluble gardenia blue pigment Z-3 is 708.1246 Da. The precursor ion ([M + Na]^+^) in the MS/MS test could offer more fragmentation information. The product ion is at *m/z* 568.0598 with the highest abundance, and it corresponded to the loss of a Δm = 163 fragment of L-Phenylalanine methyl ester hydrochloride, it is similar to water-insoluble gardenia blue pigments Z-1 and Z-2. With the loss of -CH_3_ (Δm = 15), further peaks simultaneously appeared at *m/z* 553.0389. The fragment ion at *m/z* 380.0172 was produced by the loss of a Δm = 173 fragment. Different from water-insoluble gardenia blue pigments Z-1 and Z-2, further peak simultaneously appeared at *m/z* 120.9898 because of ring cleavage, and it corresponded to the loss of a Δm = 259 fragment. The fragmentation pattern of water-insoluble gardenia blue pigment Z-3 is shown in Figure 8a. The structure of water-insoluble gardenia blue pigment Z-3 can be deduced by the functional substituents and the cleavage of the basic skeleton of the product. In this way, we can deduce that the chemical formula of water-insoluble gardenia blue pigment Z-3 is C_41_H_44_N_2_O_9_. The planar and 3D molecular structure simulation graphs are shown in Figure 8b,c.

In the first-order MS of water-insoluble gardenia blue pigments Z-4–Z-6, the [M + Na]^+^ ions at *m/z* 699.2772, 1068.4367, and 705.0953, were observed as the base peaks which prove that the formula weights of them are 676.2874, 1045.4469, and 682.1055 Da. The [M + Na]^+^ ions were voted as the precursor ion in the MS/MS test that could give more fragmentation information. The fragment ions of water-insoluble gardenia blue pigments Z-4–Z-6 were at *m/z* 536.0379, 373.9934, 120.9894, *m/z* 1036.1850, 1006.1770, 847.1269, 739.0832, 605.0628, 549.0437, 364.0243, 120.9891, and *m/z* 616.5550, 542.0380, 364.0250, 120.9898 respectively. Since the decomposition processes of water-insoluble gardenia blue pigments Z-4–Z-6 are different from the water-insoluble gardenia blue pigments Z-1–Z-3, and there are no published papers saying much, the structures, fragmentation pattern, and chemical formulas of water-insoluble gardenia blue pigments Z-4–Z-6 have not been deduced yet.

### 3.8. Solubility Study of Gardenia Blue Pigment

The results of the solubility of water-soluble and water-insoluble gardenia blue pigments in different solvents are shown in Table 7. It is concluded that the water-insoluble gardenia blue pigment is not soluble in water. Whereas the water-insoluble gardenia blue pigment can be dissolved in highly polar organic solvents, for example, acetonitrile, methanol, ethanol, acetone, ethyl acetate, and chloroform, it is not dissolved in weak polar organic solvents, for instance, ethyl ether, and petroleum ether.

### 3.9. Stability Study of Gardenia Blue Pigment

**Figure 9:**
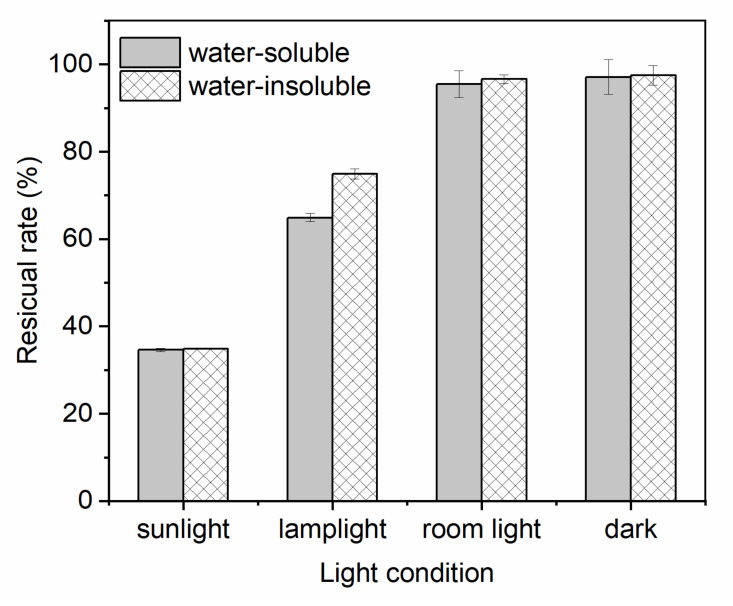
Light stability of water-soluble and water-insoluble gardenia blue pigments (residual rate).

To investigate the light stability of water-soluble and water-insoluble gardenia blue pigments, the experiments were carried out under four different light conditions: sunlight (40,000 lux), lamplight (13,000 lux), room light (300 lux), and dark (control) for 7 days. The residual rate of the pigments is shown in Figure 9.

It was found that the stability of water-insoluble gardenia blue pigment under lamplight was significantly better than that water-soluble gardenia blue pigment (*p* < 0.05), and the residual rate of it was 74.9%. However, there were no obvious differences between the water-soluble gardenia blue pigment and water-insoluble gardenia blue pigment under the light conditions: sunlight, room light, and dark (*p* > 0.05). Furthermore, the results of color properties (Table 8) were consistent with the residual rate. Under the lamplight condition, there was an apparent difference of the $\Delta L^{*},\;\Delta a^{*},\;\Delta b^{*},\;\Delta C_{ab}^{*},\;\mathrm{and}\;\Delta E_{Lab}^{*}$ between the two pigments (*p* < 0.05), the stability of water-insoluble gardenia blue pigment was significantly better than water-soluble gardenia blue pigment. However, there were no obvious differences of $\Delta L^{*},\;\mathrm{and}\;\Delta E_{Lab}^{*}\;$ between the water-soluble gardenia blue pigment and water-insoluble gardenia blue pigment under the light conditions: sunlight, room light, and dark (*p* > 0.05).

In previous studies, Zhang (2008a) studied the light stability of the hydrophobic gardenia blue pigment, which was made from the esterification of acetic anhydride and water-soluble gardenia blue pigment. The result showed that the residual rate of the pigment was 52.8% under 12,000 lux for 28 days.

**Figure 10:**
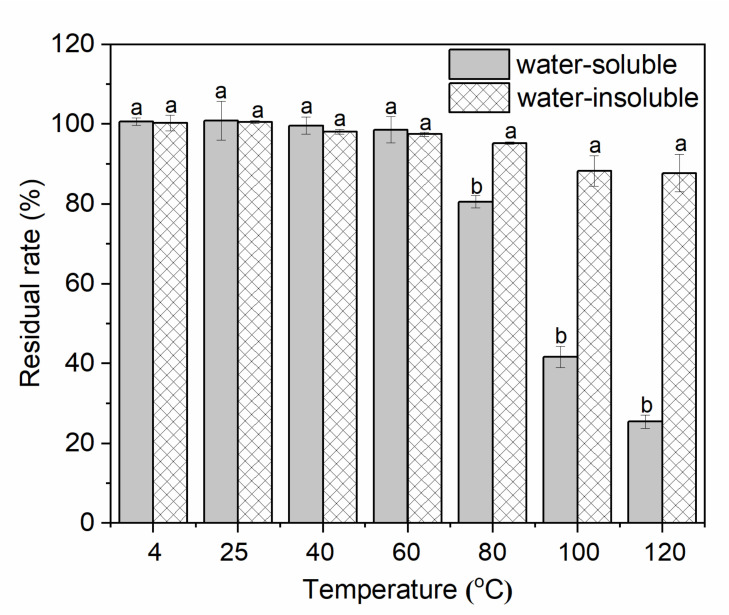
Temperature stability of water-soluble and water-insoluble gardenia blue pigments (residual rate). Different latters mean there is a significant difference (*p* < 0.05).

To investigate the temperature stability of water-soluble and water-insoluble gardenia blue pigments, the experiments were carried out under different heat conditions: 4 °C, 40 °C, 60 °C, 80 °C, 100 °C, and 120 °C, and room temperature (control) for 24 h. The residual rates of the pigments are shown in Figure 10.

It was found that the stability of water-insoluble gardenia blue pigment under 80–120 °C was significantly better than that water-soluble gardenia blue pigment (*p* < 0.05), and the residual rates of it were 95.26, 88.27, and 87.72% respectively. However, there were no obvious differences between the water-soluble gardenia blue pigment and water-insoluble gardenia blue pigment under 4–60 °C (*p* > 0.05). Furthermore, the results of color properties (Table 9) were consistent with the residual rate. Under 60–120 °C as there was an apparent difference regarding the $\Delta L^{*},\;\Delta a^{*},\;\Delta b^{*},\;\Delta C_{ab}^{*},\;\mathrm{and}\;\Delta E_{Lab}^{*}\;$ between the two pigments (*p* < 0.05), and the stability of water-insoluble gardenia blue pigment was significantly better than water-soluble gardenia blue pigment.

## 4. Conclusions

Based on molecular simulations, the synthetic route of water-insoluble gardenia blue pigment was prepared by the reaction of genipin and L-Phenylalanine methyl ester hydrochloride. After extraction and purification, we got a highly purified water-insoluble gardenia blue pigment with a high color value of 288. The result of SEM showed that the surface of the pigment was largely smooth and spherical. The UV absorbance wavelength showed that the *λ*_max_ of the pigment was 607 nm in ethanol, and the main functional groups include O-C=O, C=O, C-N, C=C, OH, and benzene ring. The results presented six different molecular weights and chemical structures of pigments, and the particular structures and formation mechanisms of three kinds of pigments were speculated (their molecular weights are 690.1156 Da, 720.1226 Da, and 708.1246 Da, respectively). The pigment was only able to be dissolved in ethanol, methanol, acetone, ethyl acetate, and other strong polar organic solvents, but was unable to be dissolved in water, ethyl ether, petroleum ether, or other weak polar organic solvents. In terms of light and thermal stabilities, water-insoluble gardenia blue pigment is significantly better than water-soluble gardenia blue pigment (*p* < 0.05). When it is under direct light for 7 days or incubated at 80–120 °C for 24 h, the pigment residual rates were 74.90, 95.26, 88.27, and 87.72%, respectively.

## Figures and Tables

**Table 1:** The results of purification of the water-insoluble gardenia blue pigment.

Purification	Recovery Ratio	Color Value	Color Value Increase	Color Properties		
				L*	a*	b*
Synthesis	/	124	/	79.32	−25.47	−14.18
Extraction	75%	219	0.77	78.99	−24.67	−15.99
Silica gel column chromatography	55%	288	1.32	78.91	−23.47	−17.47

**Table 2:** The surface functional group content of carbon element (C) of water-insoluble gardenia blue pigment.

Functional Group	E_b_ (eV)	Area (%)
π-π*	290.60	3.31
O-C=O	288.79	11.14
C=O	287.78	3.02
COOC	287.00	7.87
C-O	286.09	20.83
C-N	285.27	9.04
C=C	284.68	21.16
Sp2	284.16	23.61

**Table 3:** The surface functional group content of oxygen element (O) of water-insoluble gardenia blue pigment.

Functional Group	E_b_ (eV)	Area (%)
O-C	531.95	45.7
O=C	533.47	54.3

**Table 4:** The surface functional group content of nitrogen element (N) of water-insoluble gardenia blue pigment.

Functional Group	E_b_ (eV)	Area (%)
C-N-C	402.24	26.11
C-N	400.57	73.89

**Table 5:** The R_f_ of different spots of water-insoluble gardenia blue pigment.

Water-Insoluble Gardenia Blue Pigment	R_f_
Z-1	0.91
Z-2	0.85
Z-3	0.63
Z-4	0.77
Z-5	0.55
Z-6	0.42

**Table 6:** Accurate mass measurements and elemental compositions of the Na^+^ adduct ions and main product ions by Q-TOF MS/MS analysis of the six different water-insoluble gardenia blue pigments Z-1-Z-6.

Pigments	[M + Na]^+^ Ion (*m/z*)	Molecular Weight (Da)	Major Fragment of [M + Na]^+^ (*m/z*)	Chemical Formula
Z-1	713.1054	690.1156	550.0521	C_41_H_42_N_2_O_8_
			387.0005	
			120.9893	
Z-2	743.1124	720.1226	580.0592	C_42_H_44_N_2_O_9_
			549.0453	
			387.0007	
			120.9893	
Z-3	731.1144	708.1246	568.0598	C_41_H_44_N_2_O_9_
			553.0389	
			380.0172	
			120.9898	
Z-4	699.2772	676.2874	536.0379	/
			373.9934	
			120.9894	
Z-5	1068.437	1045.447	1036.185	/
			1006.177	
			847.1269	
			739.0832	
			605.0628	
			549.0437	
			364.0243	
			120.9891	
Z-6	705.0953	682.1055	616.555	/
			542.038	
			364.025	
			120.9898	

**Table 7:** Solubility of water-soluble and water-insoluble gardenia blue pigments.

Pigments	Water-Soluble	Water-Insoluble
Water	√	×
Dimethyl sulfoxide (DMSO)	√	√
Acetonitrile	×	√
Methanol	×	√
Ethanol	×	√
Acetone	×	√
Ethyl acetate	×	√
Chloroform	×	√
Ethyl ether	×	×
Petroleum ether	×	×

Note. √, easily soluble; ×, insoluble.

**Table 8:** Light stability of water-soluble and water-insoluble gardenia blue pigments (color properties).

Light Condition	Pigment	Color Properties				
		$\Delta L^{*}$	$\Delta a^{*}$	$\Delta b^{*}$	$\Delta C_{ab}^{*}$	$\Delta E_{Lab}^{*}$
Sunlight (40,000 lux)	Water-soluble	24.41 ± 0.66 ^a^	10.80 ± 0.19 ^b^	24.16 ± 0.22 ^a^	−26.46 ± 0.28 ^a^	36.00 ± 0.24 ^a^
	Water-insoluble	24.80 ± 0.54 ^a^	24.61 ± 0.43 ^a^	15.08 ± 0.68 ^b^	−27.76 ± 0.80 ^a^	38.05 ± 0.91 ^a^
Lamplight (13,000 lux)	Water-soluble	9.80 ± 0.30 ^a^	6.82 ± 0.16 ^a^	11.44 ± 0.50 ^a^	−13.18 ± 0.53 ^b^	16.54 ± 0.59 ^a^
	Water-insoluble	6.84 ± 0.01 ^b^	3.78 ± 0.08 ^b^	2.30 ± 0.13 ^b^	−4.34 ± 0.14 ^a^	8.15 ± 0.06 ^b^
Room light (300 lux)	Water-soluble	0.66 ± 0.01 ^a^	2.31 ± 0.18 ^a^	0.96 ± 0.01 ^a^	−1.78 ± 0.06 ^b^	2.59 ± 0.15 ^a^
	Water-insoluble	−0.39 ± 0.13 ^a^	0.89 ± 0.04 ^b^	−0.96 ± 0.01 ^b^	−0.01 ± 0.03 ^a^	1.36 ± 0.06 ^a^
Dark (control)	Water-soluble	0.32 ± 0.13 ^a^	1.73 ± 0.19 ^a^	0.53 ± 0.07 ^a^	−1.28 ± 0.14 ^b^	1.80 ± 0.21 ^a^
	Water-insoluble	−0.85 ± 0.11 ^a^	0.87 ± 0.03 ^b^	−1.00 ± 0.01 ^b^	0.03 ± 0.03 ^a^	1.58 ± 0.06 ^a^

Note. The same letter means there is no obvious difference (*p* > 0.05); The different letters mean there is a significant difference (*p* < 0.05).

**Table 9:** Temperature stability of water-soluble and water-insoluble gardenia blue pigments (color properties).

Temperature Condition	Pigment	Color Properties				
		$\Delta L^{*}$	$\Delta a^{*}$	$\Delta b^{*}$	$\Delta C_{ab}^{*}$	$\Delta E_{Lab}^{*}$
4 °C	water-soluble	0.24 ± 0.06 ^a^	−0.51 ± 0.04 ^a^	−0.20 ± 0.01 ^a^	0.08 ± 0.44 ^a^	0.60 ± 0.06 ^a^
	water-insoluble	−1.00 ± 1.39 ^a^	0.91 ± 1.08 ^a^	0.96 ± 1.17 ^a^	−1.45 ± 1.79 ^a^	1.67 ± 2.09 ^a^
25 °C (control)	water-soluble	1.41 ± 0.02 ^a^	0.11 ± 0.03 ^a^	0.74 ± 0.09 ^a^	−1.27 ± 0.70 ^a^	1.59 ± 0.06 ^a^
	water-insoluble	0.14 ± 0.01 ^b^	0.25 ± 0.06 ^a^	−0.06 ± 0.07 ^b^	−0.08 ± 0.18 ^a^	0.44 ± 0.16 ^b^
40 °C	water-soluble	0.40 ± 0.03 ^a^	1.30 ± 0.01 ^a^	0.79 ± 0.05 ^a^	−1.86 ± 0.86 ^a^	1.57 ± 0.04 ^a^
	water-insoluble	−1.22 ± 1.10 ^a^	1.22 ± 0.79 ^a^	−0.98 ± 1.27 ^a^	−1.84 ± 1.98 ^a^	2.03 ± 1.74 ^a^
60 °C	water-soluble	0.75 ± 0.11 ^a^	3.21 ± 0.16 ^a^	2.36 ± 0.36 ^a^	−3.68 ± 0.15 ^b^	4.06 ± 0.11 ^a^
	water-insoluble	0.07 ± 0.06 ^a^	1.32 ± 0.47 ^b^	0.51 ± 0.11 ^b^	−0.81 ± 1.14 ^a^	1.32 ± 0.62 ^b^
80 °C	water-soluble	7.66 ± 0.46 ^a^	1.55 ± 0.59 ^a^	4.38 ± 0.85 ^a^	−5.00 ± 0.01 ^a^	8.99 ± 0.08 ^a^
	water-insoluble	−1.05 ± 0.05 ^b^	3.21 ± 0.33 ^a^	1.45 ± 0.21 ^b^	−4.67 ± 1.48 ^a^	3.67 ± 0.38 ^b^
100 °C	water-soluble	18.04 ± 1.34 ^a^	17.20 ± 0.30 ^a^	26.78 ± 0.31 ^a^	−29.27 ± 0.60 ^b^	36.59 ± 1.03 ^a^
	water-insoluble	−2.70 ± 0.45 ^b^	9.80 ± 0.57 ^b^	6.05 ± 0.50 ^b^	−10.61 ± 1.72 ^a^	13.07 ± 1.12 ^b^
120 °C	water-soluble	26.61 ± 1.58 ^a^	19.43 ± 0.95 ^a^	35.45 ± 1.24 ^a^	−32.69 ± 0.10 ^b^	48.39 ± 2.15 ^a^
	water-insoluble	−4.51 ± 1.26 ^b^	11.67 ± 1.08 ^b^	8.95 ± 0.50 ^b^	−13.90 ± 1.47 ^a^	15.43 ± 0.16 ^b^

Note. The same letter means there is no obvious difference (*p* > 0.05); The different letters mean there is a significant difference (*p* < 0.05).

## Data Availability

The datasets used and/or analyzed during the current study are available from the correspond-ing author on reasonable request.
